# The germline genetic component of drug sensitivity in cancer cell lines

**DOI:** 10.1038/s41467-018-05811-3

**Published:** 2018-08-23

**Authors:** Michael P. Menden, Francesco Paolo Casale, Johannes Stephan, Graham R. Bignell, Francesco Iorio, Ultan McDermott, Mathew J. Garnett, Julio Saez-Rodriguez, Oliver Stegle

**Affiliations:** 1 Oncology Innovative Medicines & Early Drug Development, AstraZeneca, Milton Science Park, Cambridge, CB4 0FZ UK; 2 European Molecular Biology Laboratory, European Bioinformatics Institute (EMBL-EBI), Wellcome Genome Campus, Hinxton, Cambridge, CB10 1SD UK; 3 Microsoft Research New England, Cambridge, MA 02142 USA; 4 Wellcome Trust Sanger Institute, Genome Campus, Cambridge, CB10 1SD UK; 5 RWTH Aachen University Hospital, Joint Research Center for Computational Biomedicine (JRC-Combine), 52074 Aachen, Germany; 6 European Molecular Biology Laboratory, Genome Biology Unit, 69117 Heidelberg, Germany; 7 Division of Computational Genomics and Systems Genetics, German Cancer Research Center (DKFZ), 69120 Heidelberg, Germany; 8 Present Address: Faculty of Medicine, Institute of Computational Biomedicine, Heidelberg University, 69120 Heidelberg, Germany

## Abstract

Patients with seemingly the same tumour can respond very differently to treatment. There are strong, well-established effects of somatic mutations on drug efficacy, but there is at-most anecdotal evidence of a germline component to drug response. Here, we report a systematic survey of how inherited germline variants affect drug susceptibility in cancer cell lines. We develop a joint analysis approach that leverages both germline and somatic variants, before applying it to screening data from 993 cell lines and 265 drugs. Surprisingly, we find that the germline contribution to variation in drug susceptibility can be as large or larger than effects due to somatic mutations. Several of the associations identified have a direct relationship to the drug target. Finally, using 17-AAG response as an example, we show how germline effects in combination with transcriptomic data can be leveraged for improved patient stratification and to identify new markers for drug sensitivity.

## Abstract (web summary)

Little is known about the contribution of germline genetic variants to cancer drug sensitivity. Here, the authors devise an approach for joint analysis of germline variants and somatic mutations, identifying substantial germline contributions to variation in drug sensitivity.

## Introduction

A central premise of personalised medicine in cancer is to use molecular signatures of the tumour to predict drug response, thereby informing treatment decisions. A tractable system for deriving the necessary predictive models are in vitro screening experiments, which have allowed for assaying the efficacy of large numbers of drugs in panels of molecularly well-characterised cell lines. Initiatives such as the Genomics of Drug Sensitivity in Cancer (GDSC)^1,2^, the Cancer Cell Line Encyclopaedia (CCLE)^3^, the Cancer Target Discovery and Development (CTD^2^)^4,5^ and the Haverty et al. study^6^ have screened hundreds of cell lines derived from a broad range of cancer types, assessing their sensitivity to different compounds (predominantly targeted therapies). By correlating molecular features across cell lines with variation in the drug susceptibility phenotype, both genetic and non-genetic biomarkers have been identified.

**Fig. 1:**
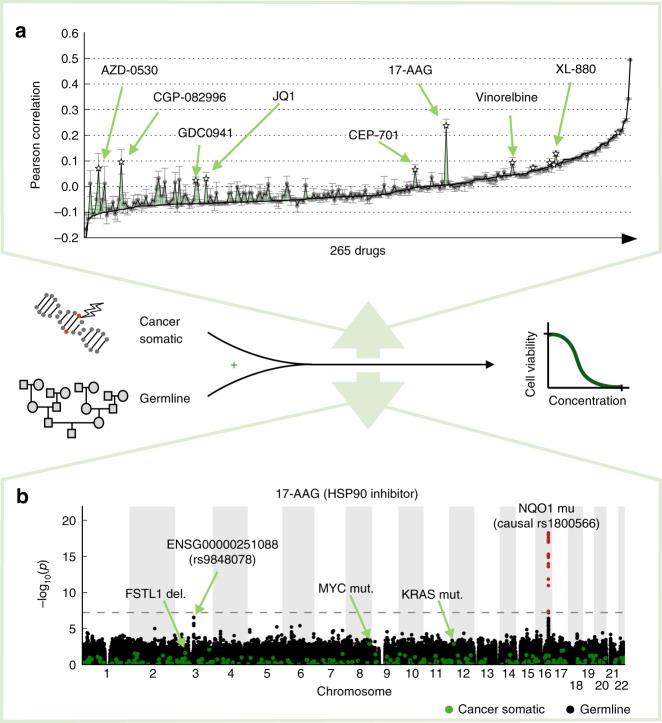
Illustration of the joint analysis approach considering germline variants and somatic mutations. **a** Prediction of drug susceptibility, either exclusively considering somatic mutations (baseline, black line) or considering the combination of germline variants and somatic mutations (green). Shown is out-of-sample prediction performance measured by the Pearson correlation coefficient between predicted and observed drug susceptibility profiles (quantified as 1-AUC; Methods). Error bars show standard deviations across analysis repetitions of the difference of Pearson correlation coefficients from the compared models (Methods, Supplementary Note 1). Selected drugs with large improvements of prediction performance when accounting for germline variants are highlighted. **b** Illustration of a joint genome-wide association analysis, considering associations between somatic mutations (green) or germline variants (black) and drug susceptibility for 17-AAG. Germline variants with genome-wide significant associations are highlighted in red (FWER < 0.05, dashed horizontal line)

Although increasingly deep molecular profiling has helped to improve the prediction of drug susceptibility^7^, genetic markers remain central for personalised treatment. This is because cancer subtypes are well characterised by the mutational profile of the tumour, but also because genetic variant data are most accessible in clinical practice. Naturally, previous analyses from in vitro screens have primarily focused on somatic changes, which can reflect causes or consequences of cancer^8^. In contrast, the relevance of inherited germline variants on drug susceptibility remains largely unknown. While individual germline variants have been associated with drug toxicity^9^, there are at-most anecdotal findings in a limited number of cancer contexts that consider both germline variants and somatic mutations to explain variation in drug sensitivity^10^. We therefore reasoned that the systematic integration of both types of genetic variations in a pan-cancer design could deliver new treatment-relevant insights, by (i) enabling improved prediction of drug susceptibility (Fig. 1a) and (ii) delivering additional germline markers for drug efficacy (Fig. 1b). The markers and mechanisms uncovered by such genetic data are clinically accessible, since the germline genetic background is stable across cells in the patient, and it can be jointly assayed with somatic mutation profiles within the same sequencing experiment.

## Results

### Identification of germline variants in cancer cell lines

We considered data from the latest revision of the GDSC screen, consisting of genetic profiles for 993 cell lines (from 30 cancer types) and drug susceptibility profiles for 265 drug compounds^2^. The GDSC project previously generated mutation profiles for 735 somatic drivers that are also observed in primary tumours (restricting to variants observed in the cancer genome atlas)^2^, including 425 recurrently copy number altered segments, 300 single-variant mutations and 10 gene fusions^2^. We reanalysed the raw genotype chip data (SNP6.0 microarray, 647,859 probes) to call germline variants, thereby extending the set of 735 somatic cancer variants. To mitigate the possibility of contamination of germline variant calls by somatic mutations, we employed statistical imputation and we assessed patterns of local linkage disequilibrium, which are expected for common inherited variants, thereby identifying likely germline variants (Methods).

### Predicting drug response from germline and somatic variants

First, we considered either somatic mutations or the combination of somatic and germline variants as input to train multivariate linear regression models of drug susceptibility (Fig. 1a). For 12 drugs, the model that accounts for germline variations yielded significantly improved prediction accuracy compared to a model based on somatic variants only (97.72% confidence interval from ten repeat experiments using fivefold cross validation of elastic net regularised linear regression; response profiles normalised by cancer type, Methods). For most of these, we observed that multiple germline variants were selected as features by the model, suggesting that the germline contribution to the response phenotype has a polygenic genetic architecture (median 44 selected variants across all drugs; Supplementary Fig. 1 and Supplementary Fig. 2). For the most striking example (17-AAG), the joint model explained 5.1% of the phenotype variance (*r* = 0.28, estimated using out-of-sample prediction), whereas a model based on somatic mutations only yielded predictions at chance level (Fig. 1a and Supplementary Data 1). Overall, the germline prediction accuracy for drugs with the largest germline component was similar to predictions based on somatic markers (Supplementary Fig. 3), including drugs with clinically approved and preclinical somatic biomarkers (e.g., *r* ≈ 0.2 for PLX4720 association with *BRAF*, selumetinib association with *KRAS* and *BRAF,* and PD-0325901 association with *NRAS* and *KRAS*).

To compare the predictive value of germline variants to data from other molecular layers, we also considered combinations of somatic mutations with gene expression profiles from the same cell lines, a commonly considered set of molecular layers^1–3,7^. In line with previous findings^2,7^, we observed that gene expression levels were a strong predictor for drug response, globally explaining larger proportions of variance than germline features. However, germline variants were as relevant or more predictive than gene expression levels for 55 drugs (~21%, Supplementary Fig. 4a, b and Supplementary Data 1). Additionally, DNA-based biomarkers are more accessible in clinical practice than gene expression^11^. We also considered a conditional analysis to account for germline signals when assessing association between gene expression levels and drug susceptibility, finding reduced associations for seven drugs (Supplementary Fig. 4c). This suggests that some of the previously reported associations between gene expression levels and drug response^1–3,7^ can in part be explainable by underlying germline effects, which were not taken into account in these analyses.

### Quantitative trait loci for drug susceptibility

**Fig. 2:**
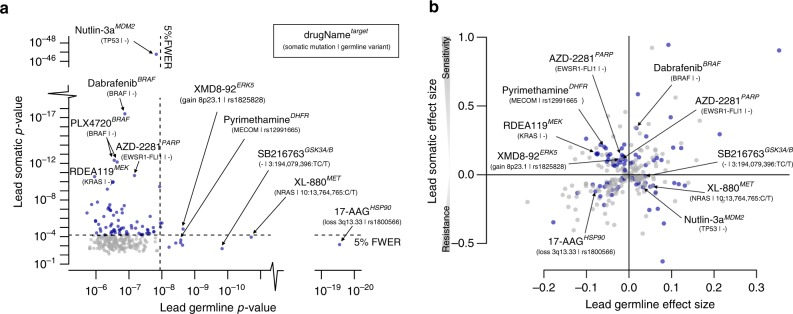
Germline and somatic associations with drug susceptibility. **a** Negative log *P* values from genome-wide association analyses with drug susceptibility phenotypes of 265 drugs, either considering lead germline variants (*x*-axis) or lead somatic mutations (*y*-axis). Shown are lead associations, i.e., the most significant association for either QTL type for each drug. **b** Effect size estimates for the associations shown in **a**, considering the corresponding lead germline associations (*x*-axis) or lead somatic associations (*y*-axis). Each dot represents a drug. Drugs coloured in blue have a significant germline or somatic association (FWER < 0.05). Somatic QTLs tended to have large effect sizes than germline QTLs. (See also Supplementary Fig. 5 for an analysis stratified by variant frequency)

Next, we used quantitative trait locus (QTL) mapping to test for genetic associations with response to each of the 265 drugs, considering both germline variants or somatic mutations. This identified 78 drugs with at least one significant drug response QTL (family-wise error rate (FWER) <5%), nine of which were associations between germline variants and drug response, including eight targeted therapies and one DNA crosslinker (Supplementary Fig. 5). As an additional quality-control step, we compared the allele frequency of germline variants that are drug response QTLs to allele frequencies observed in human reference populations (1000 Genomes Project^12^, Supplementary Table 1), finding overall consistent results. For three of nine drugs with a germline QTL, we observed a second independent somatic QTL (Fig. 2a and Supplementary Data 1). Although globally, somatic QTLs tended to have larger effect sizes than germline QTLs (average 0.125 ± 0.008 versus 0.049 ± 0.003, *P* < 10^−15^, *t*-test, Fig. 2b), both QTL types exhibited comparable effect sizes when stratifying by variant frequency (Supplementary Fig. 6). We also compared the effect sizes of germline QTLs to those observed for clinically approved somatic biomarkers, finding overall comparable effects in this in vitro cell line model (Supplementary Data 2 and 7). Finally, we assessed previously reported associations between individual germline variants and drug efficacy in vivo^13^ (Supplementary Data 3). We considered 35 individual variant–drug response pairs (nine unique drug/gene pairs) that could be assessed using our data (drug in our screen, variant allele frequency at least 2%), ten of which could be replicated (FDR < 20%), including *BRCA1/2* loss of function (LOF) with olaparib^14^ and cisplatin^15^, *WFS1* variants and cisplatin toxicity^16^, and *DPYD* LOF 5-fluorouracil^17^. Taken together, these results indicate that our germline QTL map allows screening for germline effects on drug efficacy, which analogously to previous applications of cell line models for identifying somatic biomarkers can serve as candidates for in vivo validation.

Next, we focused on the set of nine germline QTLs that reached genome-wide significance (Fig. 2a, Supplementary Fig. 8, Table 1 and Supplementary Data 1), all but the response QTL for 17-AAG^18^, to our knowledge, had not been previously observed. To assess the validity of these associations, we considered independent screening data in CCLE and CTD^2^ for replication. These screens contained the corresponding germline variant and drug response profiles for three germline–drug associations (17-AAG in both CCLE and CTD^2^, XL-880 in CTD^2^ and mytomicin in CTD^2^), all of which could be replicated (adj. *P* < 0.05, linear regression LR test) (Supplementary Table 2 and Supplementary Fig 9, Methods). We also considered a more stringent validation strategy, considering only fully independent lines that are not contained in GDSC for replication. At this reduced sample size (Supplementary Fig. 10), only the drug response QTL for 17-AAG was significant; however, we observed consistent effect size estimates between discovery and replication studies for all associations (Supplementary Table 2 and Supplementary Fig. 9). Next, we explored potential functional mechanisms of these associations. We assessed co-localisation of drug response QTL with gene expression quantitative trait loci, both using expression data in the same GDSC lines, or using data from the genotype tissue expression (GTEx) project^19^ (Methods), which identified two instances of colocalization (Supplementary Fig. 11 and Supplementary Data 4). For example, the intergenic variant rs56291722, which was associated with response to the CDK4 inhibitor CGP-082996, is also an expression quantitative trait locus (eQTL) for *GJA1* in two GTEx tissues (Nerve Tibial, Oesophagus Mucosa, Supplementary Fig. 11), suggesting that GJA1 may act as a potential mediator of this genetic effect on drug response. GJA1 (also known as Connexin43) is involved in gap junctions between cells, and has been shown to act as suppressor of metastasis from mammary tumour to lung^20^, as tumour suppressor in colorectal cancer^21^, and it is associated with reduced metastasis and cell proliferation of melanoma^22^.

**Table 1:** Associations between germline variants and drug response

Candidate causal variant	Variant annotation	Drug name	Putative drug target	Compound clinical stage	*P* value	Adjusted *P* value	Effect size
Causal rs1800566 (lead rs12595927)	Missense variant NQO1	17-AAG	HSP90	In clinical development	2.80·10^-20^	3.77·10^-5^	−7.89·10^-2^
rs67038646	Intron variant FRMD4A	XL-880	MET	In clinical development	1.94·10^-11^	1.13·10^-4^	5.00·10^-2^
rs148617501	3′ UTR variant	SB 216763	GSK3A, GSK3B	Experimental	1.48·10^-10^	1.02·10^-3^	3.84·10^-2^
rs6461564	Intron variant SP4	Mitomycin C	DNA crosslinker	Clinically approved	2.27·10^-9^	1.26·10^-2^	−5.44·10^-2^
rs1825828	Intergenic variant	XMD8-92	MAP2K5 (ERK5)	Experimental	2.36·10^-9^	1.29·10^-2^	−2.08·10^-2^
rs12991665	Intron variant DPP10	Pyrimethamine	DHFR	Clinically approved	2.66·10^-9^	1.43·10^-2^	−5.81·10^-2^
rs56291722	Downstream gene variant (eQTL for GJA1)	CGP-082996	CDK4	Experimental	6.04·10^-9^	2.98·10^-2^	3.90·10^-2^
rs7919642	Intron variant CAMK1D	AZD-0530	SRC, ABL1	In clinical development	1.09·10^-8^	4.94·10^-2^	2.10·10^-2^
rs11710820	Intergenic variant	Vorinostat	HDAC class I, IIa, IIb, IV	Clinically approved	1.10·10^-8^	4.98·10^-2^	−8.53·10^-2^

Shown are candidate causal variant for nine drugs with significant germline drug response QTLs. For all instances, but 17-AAG, shown is the lead variant; For 17-AAG, the causal variant in *NQO1* is known^52^. Variant annotations were obtained from the variant effect predictor^53^. For each drug, shown is the putative target and the clinical stage

For other germline QTLs, the identified germline variant was directly located within genes with plausible molecular mechanisms, including the association of pyrimethamine response to a germline intron variant in *DDP10* (rs12991665, Supplementary Data 1 and Supplementary Fig. 8f). Pyrimethamine targets dihydrofolate reductase (DHFR), which plays a role in DNA synthesis. DPP10 changes the biochemical properties of voltage-gated potassium channels, and in turn potassium has been reported to affect the drug target DHFR activity^23^, which is a plausible mechanism for this association.

### Molecular mechanisms of a germline QTL for 17-AAG response

**Fig. 3:**
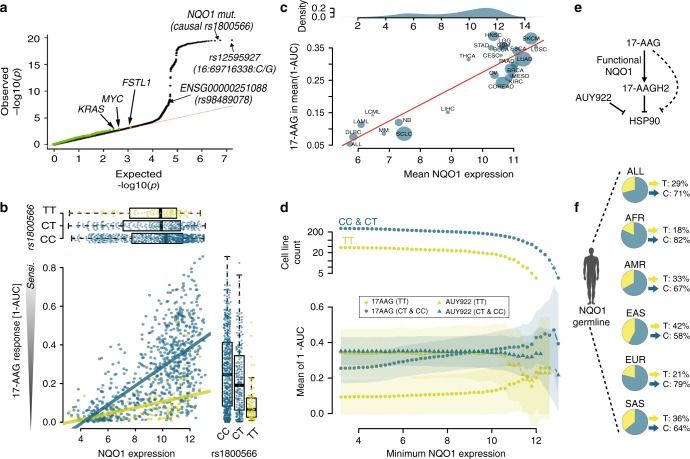
The germline component of 17-AAG drug susceptibility. **a** Quantile–quantile plot of negative log *P* values from genome-wide association tests of 17-AAG susceptibility, considering germline variants (back) and somatic mutations (green). The most associated (lead) variant rs12595927 is in tight linkage disequilibrium with the known causal variant for this associations (rs1800566, e.g., ref.^18^, *r*^2^ = 1 in European populations). **b** Scatter plot between *NQO1* gene expression (*x*-axis) and 17-AAG drug susceptibility (*y*-axis). Dots correspond to individual cell lines stratified by genotype at the rs1800566 locus (yellow: TT allele, blue: CC/CT allele). Box plots show the effect of the rs1800566 locus on gene expression (top panel) and drug susceptibility (right panel). Whereas *NQO1* expression level is not associated with the germline variant, rs1800566 modulates the association between *NQO1* expression level and drug susceptibility. The combination of high expression levels of *NQO1* together with a CC or CT genotype is associated with the largest drug response. Boxes extend from the lower quartile (Q_1_) of the data to the upper quartile (Q_2_) of the data, whiskers show the range of the data (after excluding outliers), fliers show outliers and the red lines show the medians. Outliers are defined by the standard condition *x* < Q_1_ −1.5(Q_2_ −Q_1_) ∨ *x* > Q_2_ + 1.5(Q_2_ −Q_1_). **c** Scatter plot between *NQO1* expression level (*x*-axis) and 17-AAG drug susceptibility (*y*-axis), stratified by tissue type. **d** Lower panel: Mean drug efficacy of AUY922 and 17-AAG as a function of *NQO1* expression and stratified for rs1800566 genotype. Colours indicated the genotype group with triangles corresponding to AUY922 response and circles denoting 17-AAG response. Shaded areas indicate plus or minus one standard error of the mean drug response. Top panel: number of cell lines in stratified groups. AUY922 is more effective than 17-AAG for low *NQO1* expression. In cell lines with a CT and CC (rs1800566) germline background and high expression of *NQO1*, the efficacy of 17-AAG is comparable or larger than AUY922. **e** Mode of action for 17-AAG and AUY922. **f** Frequency of *NQO1* germline variants in different human populations. Data for rs1800566 extracted from EnsEMBL

The most significant germline QTL was observed for 17-AAG response (*P* = 5.41·10^−19^, linear regression LR test, explained variance 7.1%, Table 1, Supplementary Data 1), the first HSP90 inhibitor (Supplementary Fig. 12A) that reached clinical trials^24^. Although multiple variants were significantly associated with 17-AAG response (Fig. 3a), prior evidence points to rs1800566 as the causal variant that underlies this association^18^, a common variant in the human population (allele frequency ~30%, Fig. 3f). This variant has previously been identified as loss-of-enzymatic activity^25,26^, and multiple studies documented its effect on functional expressed *NQO1* and 17-AAG efficacy^1,3,27–29^. This non-synonymous variant in the coding sequence of *NQO1*^*C609T*^ (C>T at nucleotide position 609) causes a structural change from proline>serine at amino acid position 186^24,30^, directly affecting the function of *NQO1*.

Leveraging the substantially larger sample size of our data compared to previous studies of 17-AAG response (*N* = 890 cell lines versus *N* = 4 in ref.^18^), we were able to dissect this *NQO1* association in further detail. In a joint analysis using gene expression data from the same cell lines, we observed a statistical interaction between the expression level of *NQO1* and the germline variant rs1800566, where high expression level in either the CC or CT germline background resulted in increased efficacy of the drug (Fig. 3b). Notably, these factors were unlinked, i.e., we found no evidence that rs1800566 affects the expression level of *NQO1* itself (Supplementary Fig. 12B–C). Instead, *NQO1* expression level was strongly associated with the tissue of origin of the cell line (Fig. 3c). The association between *NQO1* expression and drug response was robustly observed across and within individual cancer types (Supplementary Fig. 12D). Taken together, our data suggest a mechanism whereby expression level of *NQO1* is the main determinant of 17-AAG drug response, with an effect that is modulated by the germline background, i.e., NQO1 needs to be functionally expressed (CC or CT germline background).

There is some, albeit limited, clinical data on the impact of *NQO1* variants on quinine-based antitumor agents such as 17-AAG^31^. While the first phase clinical trial of 17-AAG also tested for germline effects due to the aforementioned variant (rs1800566)^32^, this study concluded that the variant does not affect drug efficacy. This apparent discrepancy with our data is most likely the result of a dramatic difference in sample size (21 patients versus 890 cell lines). Moreover, Goetz et al.^32^. considered the CT genotype as loss-of-function event, whereas our data suggest that the homozygous TT background only affects NQO1 function (Fig. 3b).

Finally, we explored how these insights could be leveraged for improved patient stratification. We compared drug susceptibility of 17-AAG with the more recent HSP90 inhibitor AUY922, which acts via an NQO1-independent mechanism^33^. While in the TT germline background, AUY922 was more effective than 17-AAG, our data indicate that 17-AAG is as effective as AUY922 in a CC or CT germline background, provided *NQO1* is highly expressed (Fig. 3d). To this end, it is worth noting that *NQO1* is frequently overexpressed in cancer cells compared to matching control tissue (Supplementary Fig. 12E and Supplementary Data 5), which could potentially be leveraged to guide the application of NQO1-directed agents^31,34^. Our results support such a strategy, and show that accounting for the germline background is critical for treatment success.

## Discussion

Our study provides a systematic characterisation of the effect of germline genetic variations on drug response in cancer cell lines. We find that joint modelling of somatic mutations and germline variants can yield substantially improved predictions of personalised drug efficacy. We also compared the predictive value of germline variants to gene expression profiles, finding that some of the predictive benefits of gene expression profiles on drug response can be explained by underlying germline effects.

Beyond predictions of drug susceptibility, we identified associations between individual germline variants and drug response profiles, reproducing previously known response variants as well identifying novel associations. For example, our data replicate associations between *BRCA1/2* LOF with olaparib^14^ and cisplatin^15^, as well as between *DPYD* LOF and 5-fluorouracil^17^, and between *WFS1* variants and cisplatin toxicity^16^ at marginal significance levels (FDR < 10%, Supplementary Data 3). At more lenient thresholds, we find suggestive evidence for additional known germline effects, including *MGMT* variants associated with temozolomide toxicity^35^, and *SLCO1B1* variants as methotrexate pharmacokinetics predictor^36,37^ (FDR < 30%, Supplementary Data 3). Although our data are consistent with a considerable number of known in vivo markers for drug response, others could not be replicated. There a number of possible explanations for these differences, including statistical power and limitations of in vitro models in general. Notably, our study is also based on a pan-cancer analysis, and hence tissue-specific signals will most likely be missed. Systematic tissue-specific analyses would be a natural extension our analysis, however will require larger sample sizes.

In addition to reproducing known associations, we identified nine genome-wide significant germline QTLs, most of which have not previously been reported (Table 1). This catalogue could be relevant for treatment decisions or to improve our understanding of drug action. Germline associations with drug efficacy can also guide drug development and drug safety, irrespective of whether the variant itself is used as a biomarker for patient stratification^38^. Despite the successful replication of several germline–drug associations using independent screening data (Supplementary Fig. 9 and Supplementary Table 2), there remain limitations of our analysis. In particular, as our analysis is based on genetic variant calls in cell lines, germline and somatic variants can only be distinguished computationally. We have considered different filters to identify germline variants that are known to segregate in human populations, with consistent allele frequencies to human reference populations (Supplementary Table 1). However, we cannot rule out that some of the signals we report may still be driven by somatic processes that act on the same loci. Thus, ultimately, the associations we have identified will require additional in vitro and in vivo validation.

Finally, we dissected a germline QTL for 17-AAG response, identifying an interaction effect between a germline variant and NQO1 expression affecting 17-AAG susceptibility, which tags a tissue-specific effect. Although the data set sizes of current studies are only beginning to permit such stratified analyses, we anticipate that the systematic identification of interactions and tissue-specific associations will open new venues for personalised models of drug sensitivities (Supplementary Fig. 13). The substantially increased search space to test for such interactions will require larger data sets. Ongoing studies using primary tumours, notably the initiatives by the International Cancer Genome Consortium (ICGC) and The Cancer Genome Atlas (TCGA), are starting to deliver data at the required scale. Additionally, organoid-based technologies enable drug screenings at large scale and in physiologically relevant contexts^39^. We anticipate that interaction analyses will become powerful tools to fully exploit these forthcoming data sets.

## Methods

### Drug response screen

The drug response data are based on Genomics of Drug Sensitivity in Cancer (GDSC) project release (http://www.cancerrxgene.org/downloads/)^1,2^. Drug responses are measured in one minus area under the drug response curve (1-AUC).

### Somatic mutation data

The somatic mutation data were generated by the GDSC project^2^ and assembled from the COSMIC database (http://cancer.sanger.ac.uk/cell_lines)^40^. This variant set was derived using whole-exome sequencing data (Agilent SureSelect/Illumina, https://www.ebi.ac.uk/ega/datasets/EGAD00001001039), Affymetrix SNP6.0 arrays (https://www.ebi.ac.uk/ega/datasets/EGAD00010000644) and breakpoint-specific primers for copy number variants and fusion genes. Multiple filters were applied in the primary data analysis, identifying a set of 715 curated somatic markers.

### Imputed germline variation set

An initial set of germline variants was derived from the Affymetrix SNP6.0 microarrays, which captures 884,110 common variants. For genetic analyses, we used statistical imputation to enhance the resolution of the germline QTL map. Imputation was carried out based on a 1000 Genomes Phase 3 reference panel^12^. We applied shapeit v2.r727^41^ with default parameters to obtain haplotype estimates separately for each chromosome. Imputation was performed using impute2 v2.3.2^42^, again with default parameters. This imputation was parallelised in intervals of ~5 Mb (or larger), such that each interval contained at least 200 variants. Low-frequency and rare variants (MAF > 2%, on the whole panel of 993 individuals), as well as variants with low-quality scores (quality score <0.9) were discarded, resulting in 8,251,755 genome-wide variants for analysis.

For drug response predictions (Fig. 2), variant resolution was no concern. Consequently, we used unimputed raw variants, again filtered by variant allele frequency (MAF > 2%, resulting in 645,752 variants). Additionally, we discarded variants that were not in local linkage disequilibrium (LD), only retaining variants that were in LD (*r*^2^ ≥ 0.4) with at least one of the closest 50 SNPs (Supplementary Fig. 14). This filter resulted in a set of 526,697 germline variants for analysis (See Supplementary Fig.14).

### Drug response prediction from germline and somatic variants

To assess the utility of germline variants for drug susceptibility prediction, we compared a baseline model using only somatic mutations with a joint model that combines both somatic mutations and germline variants. Both models were implemented using elastic net regularised multivariate linear regression^43^. The somatic model was trained on the set of 735 somatic variants. In order to train the joint model, we first regressed out the predictions from the somatic baseline model on the training samples, and then trained an elastic net on the residuals of the drug response phenotype, considering the set of 526,697 germline variants. Predictions from the joint model on test samples were obtained as the sum of the predictions from the baseline model and the model trained on residuals using germline variants. Prior to analysis, we regressed out tissue covariates from all drug response phenotypes and standardised all genetic features (somatic and germline). The predictive performance of both models was assessed using fivefold cross validation (under this scheme, all model hyperparameters were optimised using a nested cross validation, strictly using training data fractions only), where we considered the Pearson correlation coefficient between the predicted and observed phenotype values. Results were averaged over ten repetitions of fivefold cross validation with different random splits of the data into different folds. Standard deviations across repeat experiments were used to determine significance levels of improvements in prediction accuracy. Drugs for which the Pearson correlation between predictions from germline variants from the joint model and observed drug responses exceeded two standard deviations were considered to have a significant germline component (97.72% confidence interval, one-sided test, 12 total, Supplementary Data 1). For full details on the method and the implementation of the joint somatic and germline regression model, see Supplementary Note 1.

### Quantitative trait loci mapping of drug susceptibility

To map somatic QTLs, we employed a linear association test, accounting for tissue of origin as fixed effect covariates. Let the susceptibility phenotype (1-AUC) of a particular drug across cell lines be, ***y***, ***x***_s_ correspond to the binary encoding of a particular somatic variant and **W** denotes the matrix of covariates. This linear model can be written as

1 $${\mathbf{y}} = {\mathbf{1}}{\mu} + {\mathbf{W}}{\mathbf{\alpha}} + {\mathbf{x}}_{\mathrm{s}}\beta _{\mathrm{s}} + {\mathbf{\psi }},{\mathrm{where}}{\kern 1pt} {\mathbf{\psi }}\sim {{N}}\left( {0,\sigma _{\mathrm{e}}^2{\mathbf{I}}} \right).$$

Here, *β*_s_ denotes the effect size of the somatic variant, **α** denotes the effect size of the tissue covariate, *μ* is the intercept and **ψ** denotes residual noise.

To test for germline associations, we considered a linear mixed model, introducing an additional random effect term to account for genetic relatedness between cell lines (samples), thereby adjusting for possible confounding due to population structure^44^. This extended model can be written as:

2 $${\mathbf{y}} = {\mathbf{1}}{\mu} + {\mathbf{W\alpha }} + {\mathbf{x}}_{\mathrm{g}}\beta _{\mathrm{g}} + {\mathbf{g}} + {\mathbf{\psi }}{\kern 1pt} {\mathrm{where}}{\kern 1pt} {\mathbf{g}}\sim {{N}}\left( {0,\sigma _{\mathrm{g}}^2{\mathbf{K}}} \right){\mathrm{and}}{\kern 1pt} {\mathbf{\psi }}\sim {{N}}\left( {0,\sigma _{\mathrm{e}}^2{\mathbf{I}}} \right).$$

Analogously to the model in Eq. (1), **x**_g_ and *β*_g_ denote the germline variant of interest and its effect size, respectively, and **K** denotes the realised relatedness matrix^45,46^.

For both association models, we considered a likelihood ratio test to assess significance of the alternative hypothesis *β*_s_≠0 or *β*_g_≠0. To improve the robustness with respect to potential outlying phenotype values, all drug susceptibility phenotypes were quantile normalised to standard Gaussian distributions prior to analysis. Effect sizes of somatic and germline genetic effects were estimated by fitting the same model on the original phenotypic scale. All genetic association models were implemented in LIMIX^47,48^.

### Multiple hypothesis testing correction in QTL mapping

Significant somatic and germline QTLs were reported at a fixed family-wise error rate thresholds, considering germline variants and somatic variants separately. At FWER = 5%, this analysis yielded 68 drugs with a significant somatic QTL only, eight drugs with a germline QTL only and four drugs with a germline and a somatic QTL. For somatic variants, FWER was controlled using a Bonferroni correction of *P* values. For imputed germline variants, we employed a method based on permutations to estimate the effective number of genome-wide tests, thereby accounting for redundant tests due to genome-wide patterns of LD. Specifically, we considered the following two-step procedure

1. for each drug, we obtained 100 genome-wide minimal *P* values using a permutation procedure (see below);
2. we pooled the minimum *P* values across all permutations and drugs (2650 *P* values) to obtain an empirical null distribution, which we used to compute empirical (adjusted) *P* values.

Following ref.^49^, genome-wide-adjusted minimal *P* values were obtained using permuted genotype data for variants on chromosome 3, thereby comparing the observed association *P* values to an empirical null distribution. Genome-wide statistics were then extrapolated by adjusting the chromosome-level statistics by the relative length of chromosome 3 compared to the whole genome (correction factor ~15.6, see also ref.^49^), assuming that the variant density and the extent of LD on chromosome 3 are representative of genome-wide trends.

The final set of germline variants were filtered to rule out contamination by underlying somatic changes. Specifically, we excluded putative germline QTLs that were not in LD with at least one other variant within 100 kb (*r*^2^ ≥ 0.4). The filtered result set included nine germline QTLs at genome-wide significance, which were considered for further analysis (Table 1).

### Overlap with eQTL from the GTEx project

For each of the nine germline QTLs, we considered association *P* values from the GTEx V6 summary statistics (7,051 tissue samples, 44 tissues) between the lead drug response QTL (or a proxy variant) and any gene in 1 Mb and tissue that was analysed in GTEx^19^. Specifically, if the lead response QTL was not contained in the GTEx data set, we considered the GTEx variant in highest linkage (maximum *r*^2^ in 1000 Genomes Phase 3) as its proxy. Variants for which no GTEx proxy variant could be identified (*r*^2^ < 0.8 for all GTEx variants) were not considered for replication.

For each drug response QTL, the *P* values of association with gene expression for different gene/tissue pairs were corrected for multiple testing using Benjamini–Hochberg adjustment (Supplementary Fig. 11a).

For each drug/gene with significant association in at least one tissue (FDR < 5%), we assess signal colocalization between drug response and expression in the tissues with signal. Instances with evidence for colocalization (Pearson correlation between –log10 *P* greater than 0.8) are shown in Supplementary Fig. 11b, c. Summary results from the co-localisation analysis are provided in Supplementary Data 4.

### Replication of known associations

We assessed 35 variant–drug associations, considering germline variants that have been identified to affect drug efficacy in vivo (Supplementary Data 3). We used ref.^13^ as the primary source to compile this association lists, which we augmented by additional known association between drug 5-Fluorouracil and DPYD LOF^17,50^, an association between SN-38 and UGT1A1 promoter variant^51,52^, *WFS1* variant and cisplatin toxicity^16^, *MGMT* variants associated with temozolomide toxicity^35^, as well as *SLCO1B1* variants as methotrexate pharmacokinetics predictor^36,37^. We assessed variant–drug pairs for replication if the drug was contained in our screen, provided that the variant allele frequency was at least 2% in our study. Each variant was tested for association with drug efficacy using the same methods as used for genome-wide association analyses (see above). We used Benjamini–Hochberg to adjust for multiple testing across drug–variant pairs, and reported associations at FDR < 20% as replicated. This identified ten variant–drug pairs that could be replicated in our data (Supplementary Data 3).

### Replication of germline associations in alternative screens

Five of the nine drugs with a germline QTL identified in the GDSC discovery data set were contained in either CCLE or CTD^2^. Germline information was taken from CCLE and mapped to lines from CTD^2^, for which no genotype information was available. We considered raw germline variants without imputation for replication. Considered were proxy variants in the replication cohort that were in proximity and in strong LD with the lead variant identified in the discovery cohort (within 100 kB, *r*^2^ > 0.7). This approach identified proxy variants for three out five QTLs, including 17-AAG (both in CCLE and CTD^2^), XL-880 (in CTD^2^) and mitomycin (in CTD^2^). We considered two alternative replication strategies: (i) validating germline–drug association using all lines and (ii) considering out-of-GDSC cell lines only. The all-lines setting includes some of the same lines that were also contained in GDSC, however with independent drug response profiles. The out-of-GDSC settings is fully independent of the discovery cohort, although at much reduced sample size due to considerable overlap between these cohorts (up to 500 cell lines; Supplementary Fig. 10). The all-lines approach allowed for replicating three out of three QTLs (adj. *P* < 0.05, adjusted for 4 tests, Supplementary Table 2). The out-of-GDSC approach only allowed for replicating the germline QTL for 17-AAG response, most likely due to the much reduced sample size (Supplementary Fig. 10). However, we observed good consistency in effect size estimates between discovery and validation cohorts, including for insignificant effects (Supplementary Table 2 and Supplementary Fig. 9).

### Code availability

All association testing analysis were performed with the LInear MIXed (LIMIX) library available at https://github.com/limix/limix. An implementation of the two-step elastic network procedure we used for the joint analysis of germline and somatic mutations is available at https://github.com/PMBio/stepwise-elnet.

### Data availability

The data used for this study are available from the GDSC repository, http://www.cancerrxgene.org/downloads/^1,2^, the COSMIC database (http://cancer.sanger. ac.uk/cell_lines)^40^, the European Genome-phenome Archive https://www.ebi.ac.uk/ega/ data sets/EGAD00001001039 and https://www.ebi.ac.uk/ega/datasets/EGAD00010000644 and the GTEx resource, https://www.gtexportal.org/home/^19^.

## Electronic supplementary material

Supplementary Information

Description of Additional Supplementary Files

Supplementary Data 1

Supplementary Data 2

Supplementary Data 3

Supplementary Data 4

Supplementary Data 5
