# Supplementary material for: The germline genetic component of drug sensitivity in cancer cell lines
Source: Nat Commun. 2018 Aug 23;9:3385. doi: 10.1038/s41467-018-05811-3 (PMC6107640; doi:10.1038/s41467-018-05811-3)
Supplement: Supplementary file 1 — Supplementary Information [file 41467_2018_5811_MOESM1_ESM.pdf]

Supplementary Information: The germline genetic component of drug sensitivity in cancer cell lines

Supplementary Tables

Drug ID	Drug name	Lead germline variant	GDSC	ALL	AFR	AMR	EAS	EUR	SAS
**1026**	17-AAG	rs12595927	0.23	0.29	0.18	0.33	0.42	0.21	0.36
**308**	XL-880	rs67038646	0.22	0.21	0.23	0.18	0.25	0.21	0.18
**1025**	SB 216763	rs148617501	0.02	0.01	0.00	0.03	0.00	0.03	0.01
**136**	Mitomycin C	rs6461564	0.41	0.42	0.38	0.42	0.61	0.35	0.35
**1164**	XMD8-92	rs1825828	0.27	0.33	0.64	0.28	0.26	0.22	0.15
**71**	Pyrimethamine	rs12991665	0.07	0.08	0.03	0.02	0.17	0.04	0.12
**54**	CGP-082996	rs56291722	0.08	0.04	0.00	0.02	0.13	0.05	0.00
**38**	AZD-0530	rs7919642	0.26	0.19	0.12	0.18	0.16	0.26	0.24
**1012**	Vorinostat	rs11710820	0.03	0.01	0.00	0.02	0.00	0.04	0.01

Supplementary Table 1 Comparison of allele frequencies of germline variants with drug response QTL.
Shown are the allele frequencies of the nine germline variants with drug response QTL, considering both the discovery panel of cancer cell lines (GDSC) and different populations of the 1000 Genomes Project (All individuals (ALL), Africans (AFR), Ad Mixed Americans (AMR), East Asians (EAS), Europeans (EUR) and South Asians (SAS)).

Drug_name	Dataset	Proxy variant	alleles	R2 (proxy)	P (discovery)	Beta (discovery)	P (all lines)	adj P (all lines)	Beta (all lines)	P (out-of-GDSC)	adj P (out-of-GDSC)	Beta (out-of-GDSC)
17-AAG	CCLE	rs1800566	A/G	0.9275	3.57E-20	0.0778	1.73E-05	**6.92E-05**	0.0423	9.57E-03	**3.83E-02**	0.0588
17-AAG	CTD2	rs1800566	A/G	0.9275	3.57E-20	0.0778	2.10E-05	**8.40E-05**	0.017	3.16E-03	**1.26E-02**	0.0353
Mitomycin C	CTD2	rs6461563	G/T	0.7961	3.96E-09	-0.0509	2.13E-03	**8.52E-03**	-0.0076	7.90E-02	3.16E-01	-0.0252
XL-880	CTD2	rs17153800	A/G	0.9543	1.10E-10	-0.0479	9.15E-03	**3.66E-02**	-0.0052	4.01E-01	1.60E+00	-0.0158

Supplementary Table 2 Replication of germline drug response QTL using independent data.
Shown are replication results for four germline drug QTL discovered in GDSC considering validation data in CCLE and CTD2 respectively. R2 (proxy) denotes the correlation coefficient between the lead variant in the discovery cohort and the proxy variant used for replication. For each association, two alternative replication strategies were considered: (i) "all lines", is based on all cell lines available in the replication studies (some of which are also in GDSC), (ii) "out-of-GDSC", is based on the subset of lines that were not contained in GDSC. Reported are replication P value (P), adjusted P value (adj P) and effect size estimates for both strategies (Beta). For reference shown are also P values and effect sizes in the discovery cohort.

Supplementary Figures

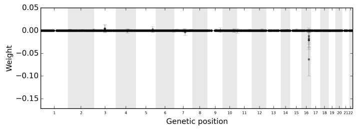

(a) 17-AAG

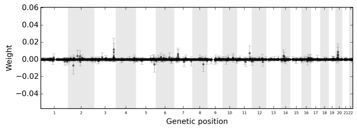

(b) CEP-701

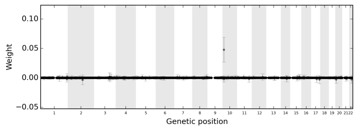

(c) XL-880

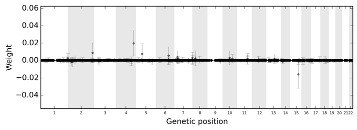

(d) Vinorelbine

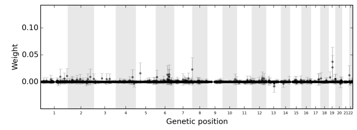

(e) AZD-0530

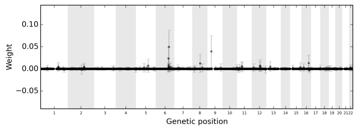

(f) CGP-082996

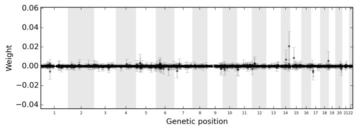

(g) JQ1

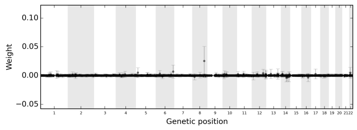

(h) GDC0941

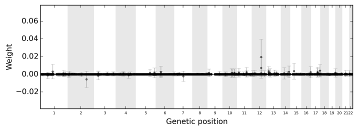

(i) YM201636

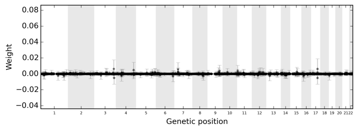

(j) Vinblastine

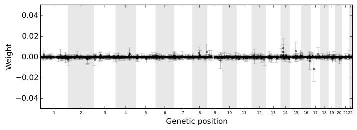

(k) Vorinostat

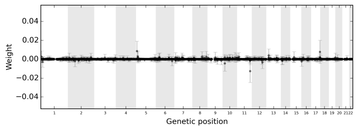

(l) PIK-93

Supplementary Figure 1 Regression weights of germline variants in drug response prediction models. Shown are averages of the regression weights from 50 training set (10 repetitions of 5-fold cross validation, ∼80% of the total dataset) with error bars indicating standard errors estimated from these repetitions. Plots show the genomic position (x-axis) versus the estimated germline variant weight (y-axis). Only drugs for which germline variants yielded significant improved drug response predictions are shown (Methods, Supplementary Data 1).

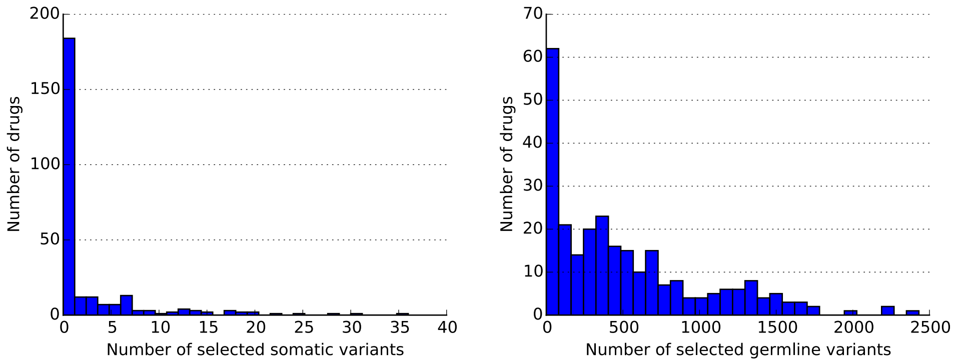

(a) somatic

(b) germline

Supplementary Figure 2 Histogram of the number of selected somatic mutations and germline variants in the joint drug response prediction model. Shown is the histogram of the number of somatic mutations (a) and germline variants (b) variants selected during training using the drug response prediction model. Individual feature were considered as selected if the average weight across 50 training sets (10 repetitions of 5-fold cross validation) was larger than the standard deviation across training sets (Methods).

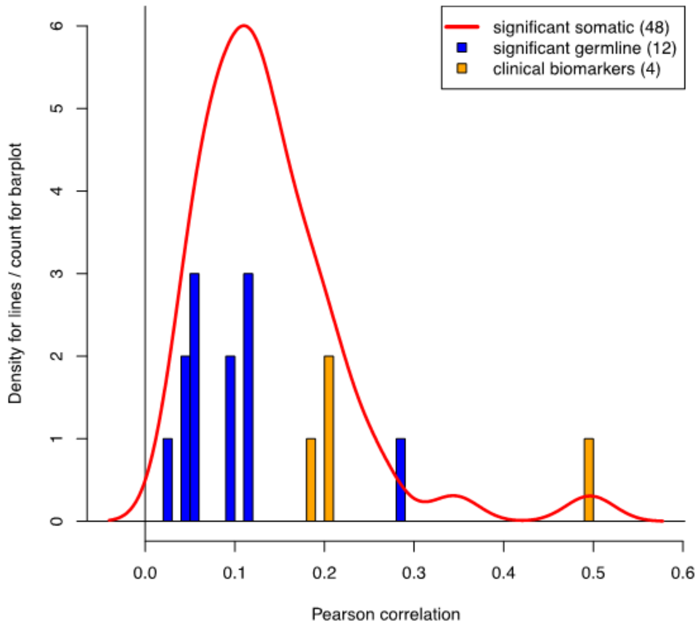

Supplementary Figure 3 Distribution of prediction accuracy of drug response profiles based on germline or somatic models. Shown is the distribution of Pearson correlation coefficients between predicted and observed drug response profiles across drugs and for alternative prediction methods. Red line: distribution of Pearson correlation coefficients based on the somatic model when considering 48 drugs with a significant somatic component. Orange histogram: Pearson correlation coefficients (somatic model) for four drugs with well-established clinical biomarkers: the two MEK inhibitors selumetinib and PD-0325901 (biomarkers: NRAS and BRAF), the BRAF inhibitor PLX4720 (biomarker BRAF) and the MDM2 inhibitor nutlin-3a (biomarker TP53 mutants). Blue histogram: Pearson correlation coefficients based on the germline prediction model for 12 drugs with a significant germline component. Shown are predictions from the germline model after conditioning on somatic variants; see Algorithm 1. Significance is assessed based on the mean and the standard deviation of the Pearson correlations estimated across 10 repeats of the cross validation procedure (Methods).

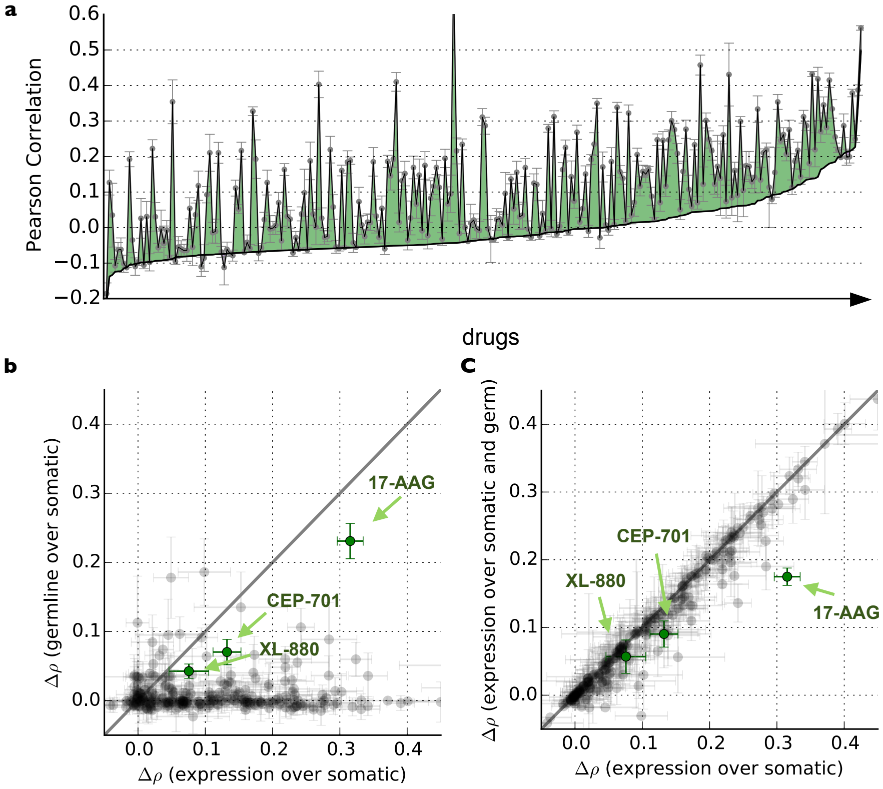

Supplementary Figure 4 Comparison of the importance of genetic features and gene expression data for predicting drug susceptibility. (a) Performance for drug susceptibility prediction, either considering exclusively somatic mutations (baseline, black) or the combination of somatic variants and gene expression (green). Prediction performance is measured as Pearson correlation coefficient between predicted and observed drug susceptibility phenotypes (1-AUC). (b) Relative improvement of prediction performance (delta Pearson correlation coefficient), comparing models that combine somatic mutations with gene expression levels (x-axis) or germline variants (y-axis) versus models that were trained using somatic mutations only. (c) Conditional analysis, assessing improvement of prediction performance when including gene expression data in addition to somatic mutations (x-axis), or when comparing to a model that contains both germline and somatic mutations (y-axis). Drugs below the diagonal indicate that gene expression levels partially tag signals that are caused by underlying germline effects. In all panels, error bars show standard deviations across analysis repetitions of the difference of Pearson correlation coefficients from the compared models (Methods, Supplementary Algorithm 1).

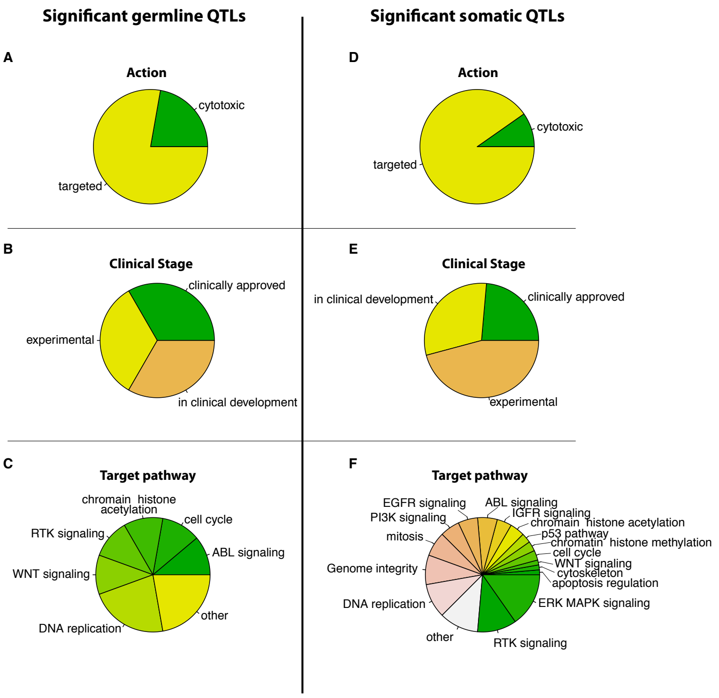

Supplementary Figure 5 Overlap of eQTL with germline drug QTL by drug category. Panels (A-C) show the drug composition for drugs with significant germline QTL (5% FWER). Panels (D-F) highlight analogous results for somatic QTL. (A,D) drugs split into cytotoxic versus targeted therapies. (B, E) clinical stages of the associated compounds. (C, F) composition of targeted pathways.

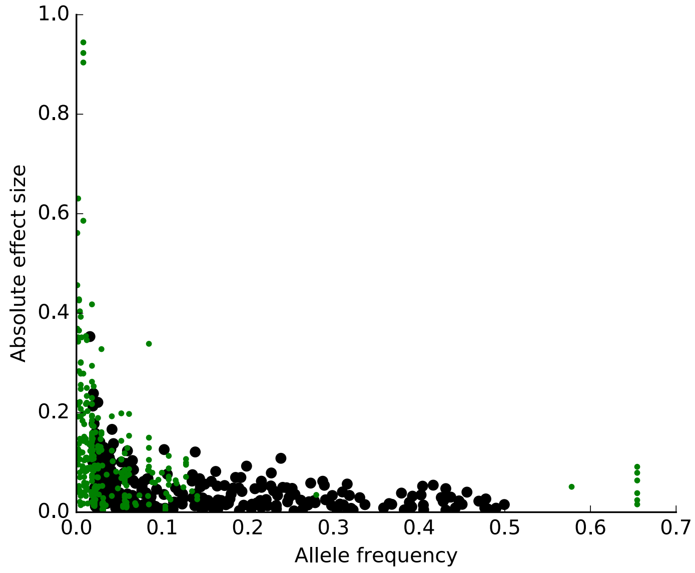

Supplementary Figure 6 Effect sizes of lead somatic and germline variants as function of the allele frequency. Shown is the variant allele frequency (x-axis) versus the absolute effect size (y-axis). Green and black dots denote somatic and germline QTL, respectively.

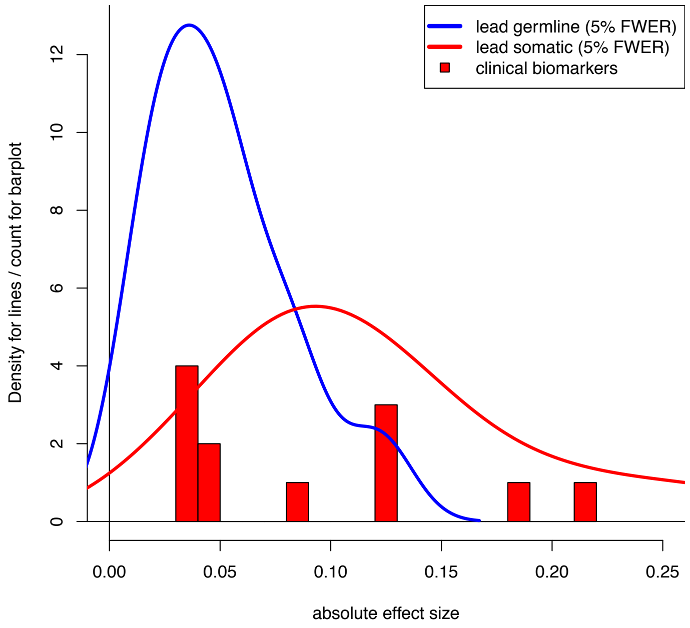

Supplementary Figure 7 Effect size distribution of germline QTL compared to somatic biomarkers. Shown is the density of absolute effect sizes for the germline and somatic QTL estimated using GDSC data in this study (blue and red line, respectively). Clinical biomarkers denote biomarkers that are i) in clinical use and ii) that were consistently detected in this dataset and replicated in the CCLE [1] (shown in red, see Supplementary Data 2).

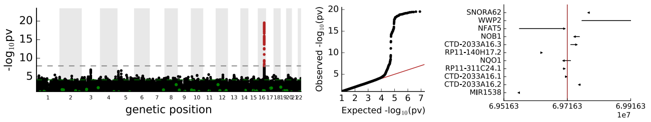

(a) 17-AAG

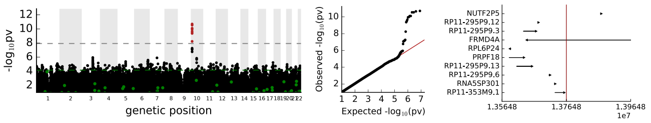

(b) XL-880

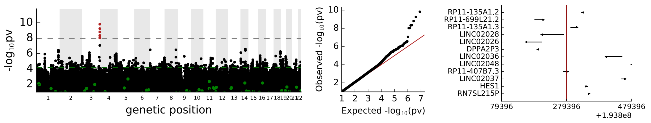

(c) SB-216763

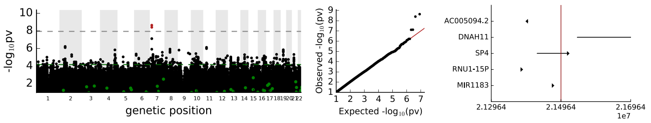

(d) Mitomycin-C

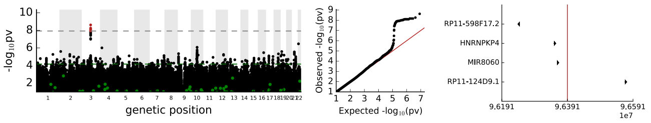

(e) XMD8-92

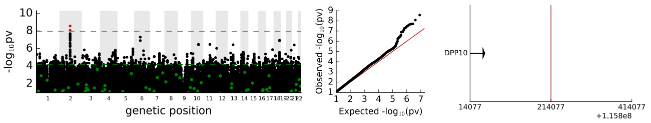

(f) Pyrimethamine

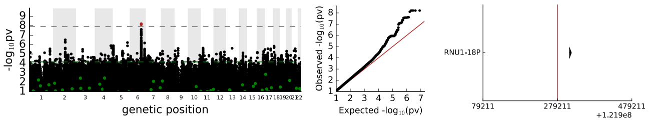

(g) CGP-082996

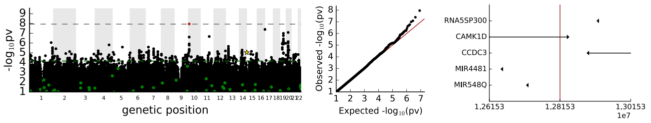

(h) AZD-0530

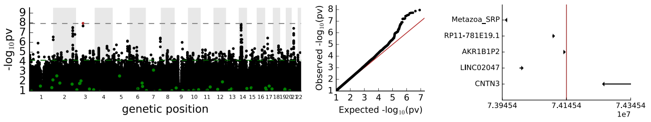

(i) Vorinostat

Supplementary Figure 8 Genome-wide Manhattan plots for drugs with significant germline QTL. Left panels: P values of genome-wide somatic mutations (green) and germline-variants (black). Middle panel: Corresponding QQ-plot. Right panel: Zoom-in view, including gene models for a 400kb region centred on the lead germline QTL variant.

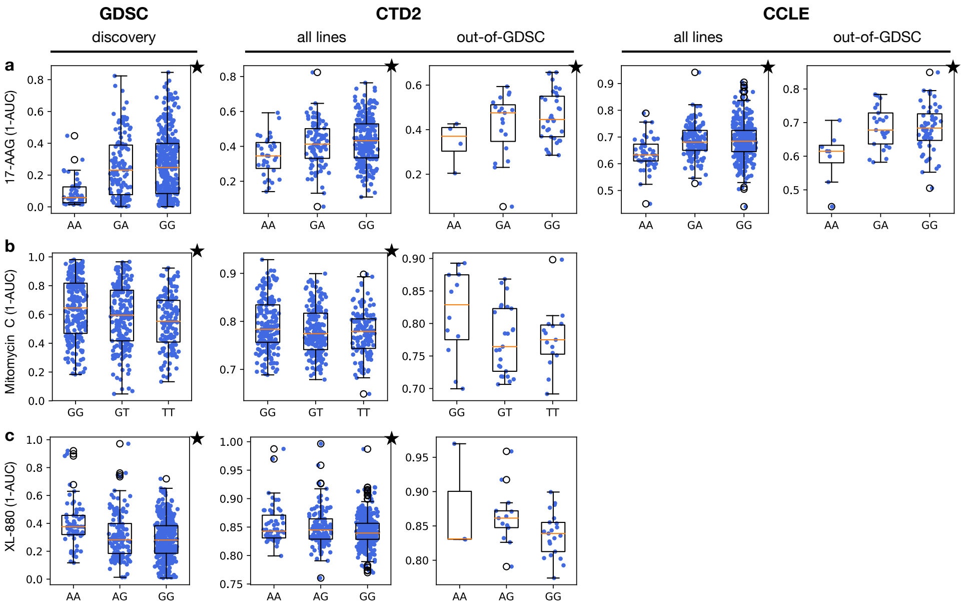

Supplementary Figure 9 Boxplots for the 3 drugs re-screened either in CTD2 or CCLE. Shown are the boxplots of drug response stratified by the germline drug response in the discovery and replication cohorts. Shown are data from two alternative validation strategies: (i) "all lines", considering all cell lines available in the validation panel (some of which are also in GDSC), (ii) "out-of-GDSC", considering the subset of lines in the validation panel that were not contained in GDSC. Significant associations are marked with an asterisk (adj $P < 0.05$). Boxes extend from the lower quartile (Q_1) of the data to the upper quartile (Q_2) of the data, whiskers show the range of the data (after excluding outliers), fliers show outliers and the red lines show the medians. Outliers are defined by the standard condition $x < Q_1 - 1.5(Q_2 - Q_1) \;\vee\; x > Q_2 + 1.5(Q_2 - Q_1)$.

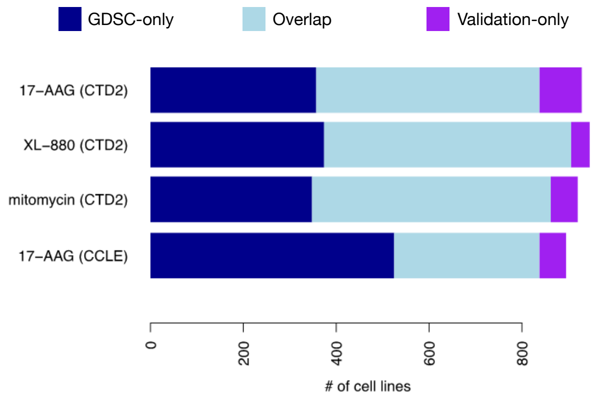

Supplementary Figure 10 Number and overlap of cell lines for the three replicable drugs across the discovery panel (GDSC) and the two validation panels (CCLE and CTD2).

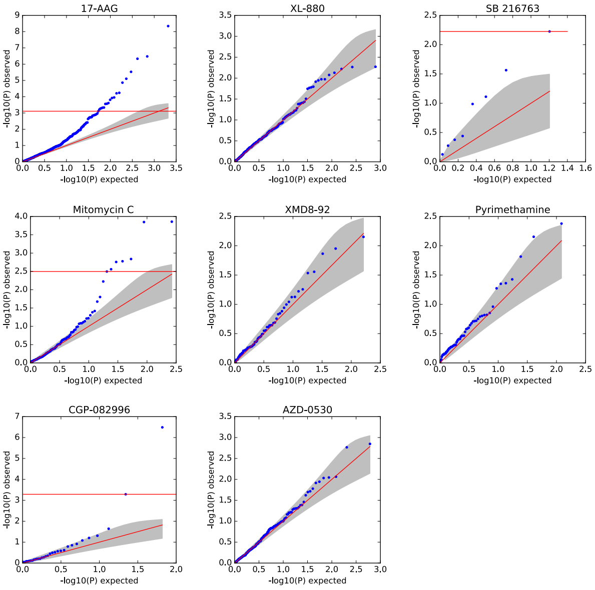

(a) somatic

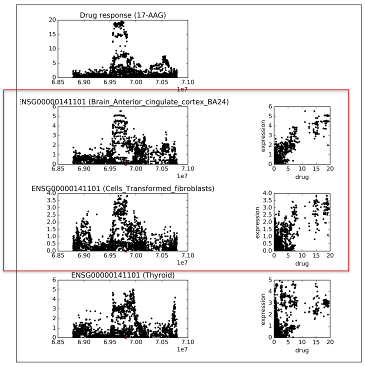

(b) 17-AAG

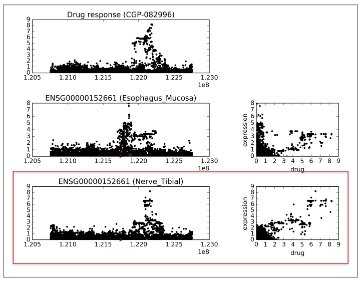

(c) CGP-082996

Supplementary Figure 11 Co-localization analysis with expression quantitative trait loci from GTEx. (**a**) For drugs with significant germline QTL, shown are QQ plots of P values for expression QTL for drug response lead variants (or a proxy variant in high LD, see Methods) for all genes within 1MB of the lead variant and across all GTEx tissues. (**b-c**) Gene/drug pairs with evidence of colocalization in at least one tissue. top panel: Manhattan plot of drug response. remaining panels (left): Manhattan plot for gene expression QTL for genes and tissues with significant eQTL at the lead germline QTL variant using data from GTEx. remaining panels (right): scatter plot of -log10 Pv of drug response (x-axis) and expression QTL (y-axis). Strong correlation indicates co-localization of both signals, meaning that the set of variants that drive both associations are shared.

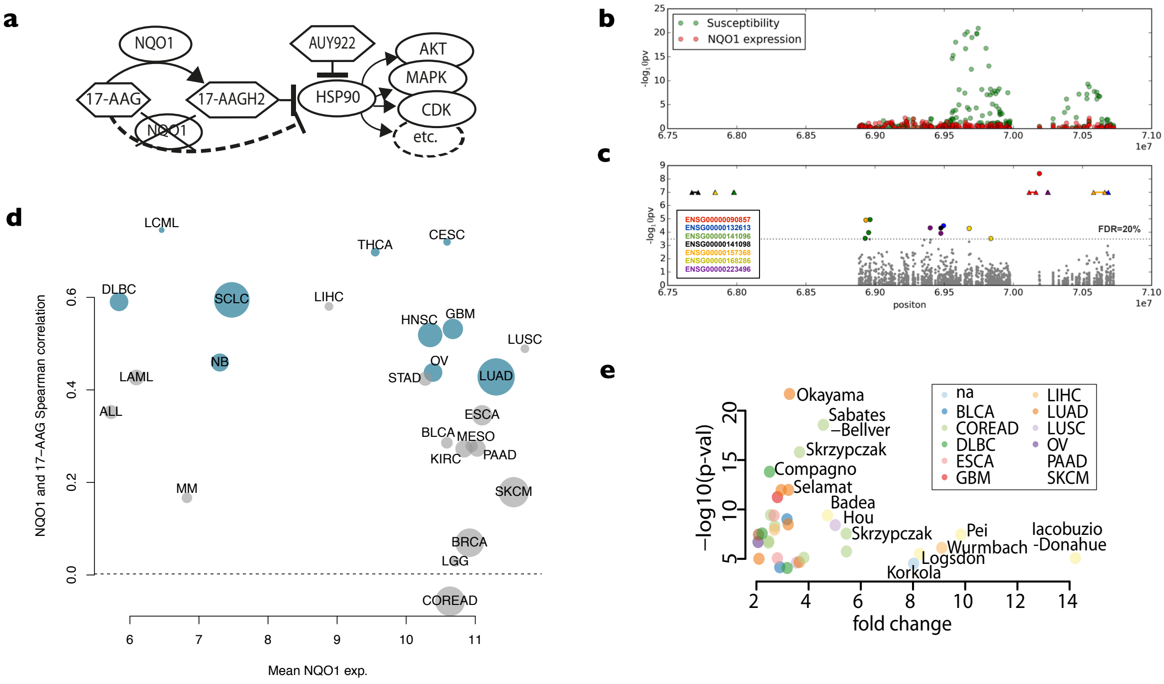

Supplementary Figure 12 Additional analyses of 17-AAG response. (**a**) Schematic of the molecular mechanism of NQO1. HSP90 is a chaperon that stabilises several client proteins including AKT, CDKs and MAPK pathway members. HSP90 can be directly inhibited through AUY-922, but also through 17-AAG. The mode of action for 17-AAG requires functionally expressed NQO1, which is metabolised into its pharmacological active form.

(**b-c**) eQTL analysis in the vicinity of NQO1. (**b**) Comparison of drug susceptibility QTL for 17-AAG (green) versus eQTL signals based on expression levels in the NQO1 expression visualised in the same Manhattan plot. (**c**) Exploring eQTL for all genes 1Mb upstream and downstream rs1800566 (i.e. the lead SNP for drug response). We also report the FDR< 20% threshold while all associated SNPs at this threshold are indicated by a coloured circle. Gene starts and ends of all genes corresponding to these associated SNPs are indicated by triangle markers (having the corresponding colour).

(**d**) Association analysis NQO1 expression stratified by tissue type. Shown area correlation coefficients between expression levels of NQO1 and 17-AAG susceptibility within various tissues. Blue dots denote significant, grey dots non-significant associations (FDR < 5%, Benjamini Hochberg adjusted). The radius of the circle is proportional to the sample size (the number of cell lines of this tissue type).

(**e**) Matched tumour versus normal expression of NQO1 for different tissue types. Shown is the fold change (x-axis) versus the p-value of differential expression (y-axis). Data obtained from TCGA; see Supplementary Data 4.

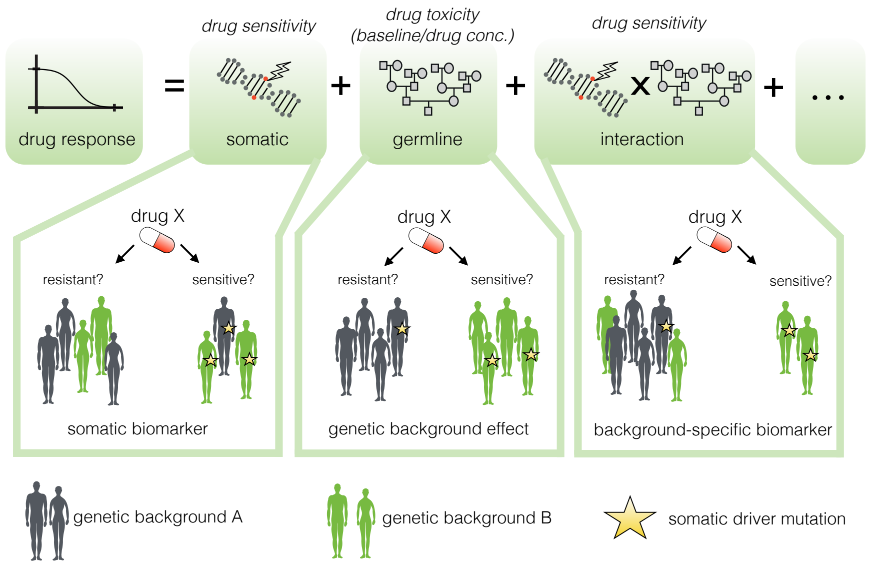

Supplementary Figure 13 Drug sensitivity as function of somatic mutations and germline variants. Schematic of drug sensitivity based on somatic mutations, germline variants and interaction effects.

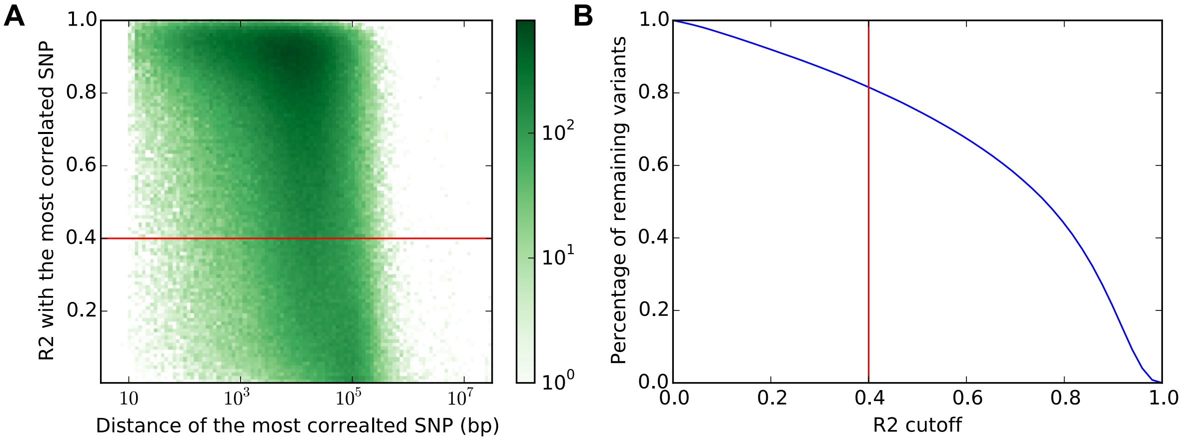

Supplementary Figure 14 Analysis of linkage-disequilibrium to verify putative germline variants. For each of the 645,752 germline variants on raw data (filtered, MAF>2%), we considered the most correlated variant among the closest 50 SNPs, as assessed using the coefficient of determination (R2). **(A)** Two-dimensional density plot of the corresponding R2 values versus the relative position for each variant in the set. Nearby variants tend to have high R2, with exceptions. **(B)** Total number of unimputed (raw) variants as a function of the minimum R2 cut-off. The red lines in both panels corresponds to R2 (R2$\geq$ 0.4), which was used for analyses presented (Methods).

Supplementary Note

Algorithms

Function `Fit-Two-Step-Elnet(`$\boldsymbol{Y}, \boldsymbol{X}^{(g)}, \boldsymbol{X}^{(s)}, K, s$`):`

Initialize-cross-validation(K, s)

for $k = 1 : K$ **do**

$\boldsymbol{Y}_{\text{train}}, \boldsymbol{X}^{(\text{s})}_{\text{train}}, \boldsymbol{X}^{(\text{g})}_{\text{train}}$ = get-training-set(k)

$\boldsymbol{X}^{(\text{s})}_{\text{test}}, \boldsymbol{X}^{(\text{g})}_{\text{test}}$ = get-feature-test-set(k)

i_{test} = get-test-set-indices(k)

fit-elnet($\boldsymbol{Y}_{\text{train}}$, $\boldsymbol{X}^{(\text{s})}_{\text{train}}$, K) //hyperparameters are optimized using inner K fold cv

$\boldsymbol{Y}^{(\text{s})}_{\star}\,[i_{\text{test}}]$ = predict-using-elnet($\boldsymbol{X}^{(\text{s})}_{\text{test}}$) //predictions from only-somatic model

$\boldsymbol{Y}_{\text{res}} = \boldsymbol{Y}_{\text{train}}$ − predict-using-elnet($\boldsymbol{X}^{(\text{s})}_{\text{train}}$)

fit-elnet($\boldsymbol{Y}_{\text{res}}$, $\boldsymbol{X}^{(\text{g})}_{\text{train}}$, K) //hyperparameters are optimized using inner K fold cv

$\boldsymbol{Y}^{(\text{g})}_{\star}\,[i_{\text{test}}]$ = predict-using-elnet($\boldsymbol{X}^{(\text{g})}_{\text{test}}$)

end

$\boldsymbol{Y}^{(\text{j})}_{\star} = \boldsymbol{Y}^{(\text{s})}_{\star} + \boldsymbol{Y}^{(\text{g})}_{\star}$ //predictions from joint model

return $\boldsymbol{Y}_{\star}^{(s)}, \boldsymbol{Y}_{\star}^{(g)}, \boldsymbol{Y}_{\star}^{(j)}$

Inputs :
$\boldsymbol{Y} = N \times 1$ vector of drug responses across N cell lines;
$\boldsymbol{W} = N \times T$ matrix of tissue type labels for N cell lines and T tissue types;
$\boldsymbol{X}^{(\text{s})} = N \times F^{(\text{s})}$ matrix of somatic variants;
$\boldsymbol{X}^{(\text{g})} = N \times F^{(\text{g})}$ matrix of germline variants;
S = number of seeds
K = number of folds

//preprocessing
$\boldsymbol{Y}$ = regressout($\boldsymbol{Y}, \boldsymbol{W}$)
$\boldsymbol{Y}$ = standardize-cols($\boldsymbol{Y}$)
$\boldsymbol{X}^{(\text{g})}$ = standardize-cols($\boldsymbol{X}^{(\text{g})}$)
$\boldsymbol{X}^{(\text{s})}$ = standardize-cols($\boldsymbol{X}^{(\text{s})}$)

//runs multiple seeds
rho_som = []; rho_germ = []; rho_joint = []
for $s = 1 : S$ **do**

$\boldsymbol{Y}^{(\text{s})}_{\star}, \boldsymbol{Y}^{(\text{s})}_{\star}, \boldsymbol{Y}^{(\text{j})}_{\star}$ = Fit-Two-Step-Elnet($\boldsymbol{Y}, \boldsymbol{X}^{(\text{g})}, \boldsymbol{X}^{(\text{s})}, K, s$)

rho_som.append(Pearson($\boldsymbol{Y}, \boldsymbol{Y}^{(\text{s})}_{\star}$))

rho_germ.append(Pearson($\boldsymbol{Y}, \boldsymbol{Y}^{(\text{g})}_{\star}$))

rho_joint.append(Pearson($\boldsymbol{Y}, \boldsymbol{Y}^{(\text{j})}_{\star}$))

end

//assessment of significance
z = mean(rho_germ) / std(rho_joint)
Significant germline component $\iff z \geq 2$
$\sigma_{\Delta\rho} = \sqrt{\text{var(rho\_joint)} + \text{var(rho\_som)}}$
//mean(rho_joint), mean(rho_som) and $\sigma_{\Delta\rho}$ are used in Fig 1a

Supplementary Note 1: Pseudocode for the joint prediction model using both germline and somatic variants. Full details of the models and methods used to assess the utility of germline variants to predict drug susceptibility. We considered cross validation (5-fold, $K =$ 5) and 10 repetitions ($S = 10$). $\boldsymbol{Y}^{(\text{s})}_{\star}, \boldsymbol{Y}^{(\text{s})}_{\star} and \boldsymbol{Y}^{(\text{j})}_{\star}$ are out-of-sample predictions from 5-fold cross validation where considering somatic variants only, germline variants only (after accounting for somatic variation) and both layers, respectively. For each repetition, Pearson correlations for the only-somatic, only-germline and joint models were computed correlating $\boldsymbol{Y}^{(\text{s})}_{\star}, \boldsymbol{Y}^{(\text{s})}_{\star} and \boldsymbol{Y}^{(\text{j})}_{\star}$ with the observed drug responses, respectively.

References

[1] Iorio, F. *et al.* A landscape of pharmacogenomic interactions in cancer. *Cell* **166**, 740–754 (2016).

[2] Asher, G., Dym, O., Tsvetkov, P., Adler, J. & Shaul, Y. The crystal structure of nad (p) h quinone oxidoreductase 1 in complex with its potent inhibitor dicoumarol. *Biochemistry* **45**, 6372–6378 (2006).
