# Supplementary material for: The germline genetic component of drug sensitivity in cancer cell lines
Source: Nat Commun. 2018 Aug 23;9:3385. doi: 10.1038/s41467-018-05811-3 (PMC6107640; doi:10.1038/s41467-018-05811-3)
Supplement: Supplementary file 2 — Description of Additional Supplementary Files [file 41467_2018_5811_MOESM2_ESM.pdf]

Description of Additional Supplementary Files

File Name: Supplementary Data 1
Description: **Results from the prediction and association analyses.** Sheet 1-2, Predictability of drug susceptibility using somatic mutations and germline variants. Provided are Pearson correlation coefficients between observed and predicted drug susceptibly values, when considering alternative models that use either germline variants, somatic mutations, gene expression levels or combinations of these features. The reported average correlation coefficients and standard deviations are estimated from 10 repetitions of 5-fold cross validation (Methods). Sheet 3, List of significant germline/somatic associations (single-variant QTL). Sheet 4, Drug details including action, pathway, clinical stage and putative target.

File Name: Supplementary Data 2
Description: **Clinical and preclinical biomarkers observed in CCLE and GDSC.** Provided are drugs and somatic biomarkers that are i) used in clinical practice and ii) show replicated effects in both CCLE and GDSC. The effect size provided were obtained using the same cell lines and methodology as used for the reported germline variant QTL.

File Name: Supplementary Data 3
Description: **Gold standards for germline QTL.** Provided are variant - drug pairs for 31 documented germline drug associations that have previously been observed in vivo and for which the drug is present in the GDSC screen used in this study.

File Name: Supplementary Data 4
Description: **Results from GTEx eQTL co-localization.** Provided are summary statistics from the GTEx co-localization analysis with the identified germline QTL.

File Name: Supplementary Data 5
Description: **Differential expression of NQO1 in cancer versus matched control tissue in patients.** Provided are significant differentially expressed genes obtained via the Oncomine software.
